# Association of employment quality with depression among sexual and gender minority adults: a retrospective cohort study

**DOI:** 10.1016/j.lana.2026.101462

**Published:** 2026-03-26

**Authors:** David J. Kinitz, Nguyen K. Tran, Faraz V. Shahidi, Shamsi Soltani, Kinsey B. Bryant-Lees, Annesa Flentje, Micah E. Lubensky, Juno Obedin-Maliver, Mitchell R. Lunn

**Affiliations:** a The PRIDE Study/PRIDEnet, Stanford University School of Medicine, Palo Alto, CA, USA; b Division of Nephrology, Department of Medicine, Stanford University School of Medicine, Stanford, CA, USA; c Department of Obstetrics and Gynecology, Stanford University School of Medicine, Stanford, CA, USA; d Institute for Work & Health, Toronto, ON, Canada; e Dalla Lana School of Public Health, University of Toronto, Toronto, ON, Canada; f Department of Epidemiology and Population Health, Stanford University School of Medicine, Stanford, CA, USA; g Department of Psychological Science, Northern Kentucky University, Highland Heights, KY, USA; h Stanford Prevention Research Center, Department of Medicine, Stanford University School of Medicine, Stanford, CA, USA; i Alliance Health Project, Department of Psychiatry and Behavioral Sciences, University of California San Francisco, San Francisco, CA, USA

**Keywords:** Sexual and gender minority, LGBTQ, Employment quality, Unemployment, Depression, Mental health

## Abstract

**Background:**

Sexual and gender minority (SGM) people face concerning rates of low-quality employment, unemployment, and depression. Our objective was to center employment in SGM mental health research and assess associations between employment quality and depression.

**Methods:**

This retrospective study used data from The PRIDE Study—a national, community-engaged, longitudinal cohort of SGM adults in the United States. Employment quality in 2021 was categorized as: standard, secure-income; standard, insecure-income; non-standard, secure-income; non-standard, insecure-income; and unemployed. Mean levels of depressive symptoms were measured using the Patient Health Questionnaire-9 (PHQ-9) from 2021 to 2023. Mixed-effects linear regression models were used to evaluate the association between employment quality and PHQ-9 scores.

**Findings:**

Participants’ (n = 3354) median age was 34.7 years; 53.1% (n = 1782) were cisgender (sexual minority), 46.9% (n = 1572) transgender and gender diverse (TGD; any sexual orientation), and 91.5% (n = 3070) were white or selected multiple race/ethnicities, including white. Participants occupied standard, secure-income (57.0%; n = 1913); standard, insecure-income (9.3%; n = 313); non-standard, secure-income (12.8%; n = 432); non-standard, insecure-income (14.9%; n = 500); and unemployed (5.9%; n = 196) groups. PHQ-9 scores were highest (*i.e.*, greater depressive symptoms) among standard, insecure-income; non-standard, insecure-income; and unemployed groups. PHQ-9 scores were higher among TGD workers in all employment quality groups compared to cisgender workers. In adjusted models, workers in standard, insecure-income (difference = 0.98; 95% CI, 0.38–1.58; p = 0.001); non-standard, insecure-income (difference = 1.39; 95% CI, 0.88–1.90; p < 0.001); and unemployed (difference = 3.12; 95% CI, 2.38–3.85; p < 0.001) groups reported higher depressive symptoms compared to the standard, secure-income group.

**Interpretation:**

Poorer employment quality was associated with higher levels of depressive symptoms among SGM workers.

**Funding:**

Dona Rockstad, 10.13039/100006093Patient-Centered Outcomes Research Institute.

**Research in context**

**Evidence before this study**

The impact of poor employment quality on the mental health of the working population has been consistently demonstrated across public health literature, particularly impacts on depression. However, sexual and gender minority (SGM) populations have been overlooked with few exceptions. This is pressing given that SGM populations face disproportionate levels of depression compared to the general population, as well as poorer employment quality. We conducted a scoping review of employment quality among SGM workers across Organization for Economic Co-operation and Development (OECD) countries (doi.org/10.1007/s13178-024.00950-3) that relied on a comprehensive, peer-reviewed search strategy (doi.org/10.33137/utjph.v3i2.37455). In addition, we searched PubMed up to July 2025 using an adapted version of the published search strategy, searching terms for SGM populations (*e.g.*, “LGBTQ” or “transgender” or “gay”), employment (*e.g.*, “work” or “occupation”), and health (*e.g.*, “anxiety” or “depression” or “wellbeing”). We identified one additional study that presented findings consistent with extant literature.

**Added value of this study**

To our knowledge, this is the first longitudinal study to evaluate the relationship of employment quality and depression among SGM populations. In addition to bringing SGM populations into occupational health literature, this study responds to calls for multidimensional definitions of employment quality in occupational health research and socioeconomic-focused research among SGM populations. Guided by prior scholarship, we used income as a key variable in our employment quality groups. Income is often omitted from employment quality and health scholarship. Importantly, our study included multiple sexual and gender minority groups with models to understand important differences by gender modality (cisgender and transgender). Gender has rarely been considered beyond binary sex comparisons in occupational health scholarship, and studies focused on SGM population health have commonly considered SGM populations as a homogenous group or only focused on cisgender sexual minority workers or gender minority workers of any sexual orientation. Further, we used a validated measure for depressive symptoms that provides increased rigor and precision compared to self-reported general mental health using a Likert scale.

**Implications of all the available evidence**

Interventions should address mental health and employment among SGM workers. Reducing the number of workers exposed to unemployment and insecure-income employment may reduce the burden of depression among SGM workers. Findings should motivate and encourage occupational health researchers to consider SGM workers, and sub-groups within the larger SGM population, as a unique demographic of workers worthy of dedicated attention in population health studies. SGM health researchers should consider employment as a central variable in SGM health inequities research.

We were unable to conduct intersectional analyses due to small cell size. The lack of population datasets that include adequate sexual orientation and gender identity variables requires dependence on community samples. Additional population-level data are needed to better understand differences in the association of mental health and employment quality among cisgender sexual minority and TGD workers and their diverse social identities. Qualitative data are also needed to further understand the mechanisms of these differences.

## Introduction

Sexual and gender minority (SGM) populations face elevated levels of depression relative to heterosexual and cisgender populations.^1^^,^^2^ In a meta-analysis of 52 studies, 18% of gay and lesbian people and 23% of bisexual people reported current binary indicators of depression compared to 13% of heterosexual individuals.^1^ Within SGM groups, depression is highest among transgender people with 33.3% having reported moderate depression in the past two weeks.^2^

Employment—whether one is employed or not—and employment quality are key determinants of depression and are associated with other social determinants of depression including housing, food security, income, social support, and healthcare access.^3^, ^4^, ^5^ Key features of employment quality that impact depression include compensation (secure *versus* insecure), contract type (permanent *versus* temporary), and status (whether one is respected/holds power at work).^6^ Good quality employment, such as standard employment (*i.e.*, full-time, stable employment often associated with adequate wages, worker protections, and health benefits), can improve depression by providing access to income and employment security, health insurance, and more equitable workplace power relations.^5^ Contrarily, low-quality employment (*i.e.*, unstable employment associated with temporary contracts, inadequate wages, and limited health benefits and worker protections) and unemployment can negatively impact depression due to income and employment insecurity, no health insurance, and a marginalized position within the workplace.^5^, ^6^, ^7^, ^8^

Contrary to public health evidence, economic policies that favored capitalist values (*e.g.*, maximizing profit at the expense of workers’ rights and health) led to a decline in standard employment and increased low-quality employment.^9^^,^^10^ These policies reduce the rights and protections for marginalized workers who are already vulnerable to labor market exploitation, resulting in a disproportionate share of SGM workers in low-quality employment and unemployment.^11^, ^12^, ^13^, ^14^, ^15^ Without secure employment with protections, SGM workers are unable to address workplace harassment and discrimination without fear of reprisal.^12^ Cisgender sexual minority workers are more likely than cisgender heterosexual workers to be in low-quality employment, while transgender and gender diverse (TGD) workers of any sexual orientation face even poorer employment outcomes than any gender group.^11^^,^^16^

Occupational and mental health have largely remained distinct research domains despite being closely linked. Minority stress, a prominent theory employed in SGM health disparities research, may be restrictive in its historical focus on interpersonal workplace contexts (*e.g.*, co–worker interactions) without attention to the broader structural employment relationships (*e.g.*, standard *versus* non-standard employment)^17^ that may ultimately impact individual-level mental health. Using a social determinants of health framework, we situated our objectives to bring these research domains together by examining employment as an important and overlooked determinant of SGM mental health.^18^^,^^19^ We further explored potential gender group differences given how gender norms may shape employment quality and depressive symptoms.^13^^,^^20^^,^^21^

Guided by prior literature and conceptual models of employment quality and mental health, we aimed to do the following: 1) To examine the association between employment quality and depressive symptoms over a three-year follow-up period. We hypothesized that participants in lower-quality employment categories—standard, insecure-income; non-standard, secure-income; non-standard, insecure-income; and unemployment—will exhibit higher PHQ-9 scores compared with those in standard, secure-income employment. 2) We aimed to assess whether the association between employment quality and PHQ-9 score changes over time. We hypothesized that differences in PHQ-9 scores between employment quality groups will worsen across the three-year follow-up period. 3) We aimed to evaluate whether gender modality modifies the association between employment quality and PHQ-9 scores. We hypothesized that the association between lower-quality employment and higher PHQ-9 scores will be stronger among transgender and gender diverse (TGD) participants compared with cisgender sexual minority participants.

## Methods

### Reflexivity statement

We occupy a diversity of genders and sexual orientations, have or have had experiences of non-standard and precarious employment, and are actively involved in SGM communities. This guides our approach to research, including research question development, data collection methods, and dissemination strategies grounded in social justice.

### Study design and sample

We analyzed data from The Population Research in Identity and Disparities for Equality (PRIDE) Study—a national, online, community-engaged cohort of SGM adults in the United States (US)—with outreach and recruitment at SGM events, word-of-mouth within SGM networks, and social media advertising.^22^^,^^23^ Enrollment criteria included: being 18 years of age and older, self-identifying as a SGM person, living in the US, and reading and understanding English. Informed consent was obtained electronically, and, upon enrollment, participants were invited to complete a self-administered lifetime and current annual health questionnaire, with invitations to complete follow-up questionnaires annually. All questionnaires are administered through The PRIDE Study's digital research platform, which was designed to mitigate automated bot responses and ensure data quality.^24^ Ethics approval was overseen by the Stanford University Institutional Review Board (IRB), University of California San Francisco IRB, and the WIRB-Copernicus Group (WCG) IRB for all ongoing analyses. This study followed the Strengthening the Reporting of Observational Studies in Epidemiology (STROBE) guidelines.

The analytic sample in this study included participant responses from three recent annual questionnaires (2021–2023 administration periods) from The PRIDE Study. We included participants who completed the 2021 questionnaire, had exposure data in 2021, and provided outcome data for at least one timepoint between 2021 and 2023. Participants were excluded if they were not active in the labor market (*i.e.,* unemployed and not looking for employment; homemaker; unable to work due to a disability; or retired), identified as students, or had missing gender identity data in 2021. Completion rates for annual questionnaires ranged from 75% to 79%.

### Measures

#### Exposure

Based on literature describing the multidimensional nature of employment quality (*e.g.,* income, employment security, worker protections), self-reported employment status and annual income in 2021 were used to create a five-group exposure of employment quality.^9^ Participants were asked: 1) “Which of the following describes your current occupation or employment status (select all that apply)?” with the following response options: employed, working 40 h or more per week [full-time]; employed, working 1–39 h per week [part-time]; temporarily employed; self-employed; and not employed, looking for work [unemployed]; and 2) “What were your individual earnings (in US dollars) before taxes and deductions from ALL sources in the 2020 tax year?” We dichotomized the response options to ≤$30,000 and >$30,000. We used a $30,000 annual income cut-off based on the concept of income inadequacy proposed in extant studies that used approximately twice the federal poverty level in 2020 as a benchmark for a low living wage in the US.^9^^,^^25^ Participants were grouped into: (i) ‘standard, secure-income’ (reported only full-time employment *and* income >$30,000); (ii) ‘standard, insecure-income’ (reported only full-time employment *and* income ≤$30,000); (iii) ‘non-standard, secure-income’ (reported part-time, temporary, self-employed, or selected multiple employment options, *and* earned >$30,000); (iv) ‘non-standard, insecure-income’ (same criteria as non-standard *and* earned ≤$30,000; and (v) ‘unemployed’ (reported only unemployed but looking for work). Participants who selected multiple employment types (*e.g.*, full-time and self-employed, full-time and temporary, full-time and part-time, unemployed but looking for work and temporary) were categorized into the non-standard group.

#### Outcome

Depressive symptoms in the past two weeks were measured at all three timepoints using the Patient Health Questionnaire-9 (PHQ-9) instrument, which includes nine depressive symptom items (scored 0–3) with a total score range of 0–27 (higher scores indicate more symptoms).^26^ A 3-point change in PHQ-9 score can be interpreted as clinically meaningful.^27^

#### Gender modality

Participants self-reported their gender identity and sex assigned at birth in 2021. Participants could select multiple responses for gender identity. We categorized gender modalities (*i.e.*, cisgender or TGD) based on gender identity and sex assigned at birth using a two-step procedure.^28^^,^^29^ Cisgender sexual minority participants were all sexual minority individuals whose gender identity was concordant with the gender commonly associated with their sex assigned at birth. TGD participants of any sexual orientation included any SGM participant from The PRIDE Study whose gender identity was discordant with the gender commonly associated with their sex assigned at birth or who endorsed any of the following current gender identities: agender, genderqueer, nonbinary, questioning, transgender man, transgender woman, Two-Spirit, and another gender identity.

#### Additional socio-demographics

Participants self-reported their ethnoracial identity, sexual orientation, immigration status, and intersex status in 2021. Participants could select multiple responses for ethnoracial identity and sexual orientation. Education level was self-reported, and age was calculated based on birth date. We categorized immigration status as non-US born and US born, and US Census region as Northeast, Midwest, South, and West based on self-reported Zone Improvement Plan (ZIP) code. Urbanicity was based on the 2010 US Department of Agriculture Rural-Urban Community Area (RUCA) codes.^30^ Urban was defined as including all metropolitan primary codes (*i.e.*, 1, 2, and 3) and non-metropolitan areas in which 30% or more commuted to a nearby metropolitan core (*i.e.*, 4.1, 5.1, 7.1, 8.1, and 10.1). All other RUCA codes were categorized as rural areas.

### Statistical analysis

We described participant characteristics for the overall sample and by employment quality, and calculated means, medians, and standard deviations of PHQ-9 scores over time, stratified by employment quality and gender modality. We used a series of mixed-effects linear regression models to evaluate the association between employment quality and PHQ-9 scores over time. In these models, all available outcome data over the 3-year period were included from participants who completed the 2021 annual questionnaire. Maximum likelihood estimation was used to handle missing outcome data assuming “missing at random,” whereas listwise deletion was used for missing covariate data.^31^ Model 1 regressed employment quality and time (years since 2021, centered at grand mean) on PHQ-9 scores. Model 2 [primary model] added grand mean-centered age, gender modality, education level, immigration status, US Census region, and urbanicity to adjust for potential confounding. Model 3 examined potential employment quality by time interaction, and model 4 explored employment quality by gender modality interaction. All models included a random intercept for participants and a linear random slope for time. We evaluated model fit using the Akaike Information Criterion, and the joint statistical significance for interaction was assessed using the Likelihood Ratio Test comparing nested models with and without interaction terms. Model diagnostics were assessed using residuals and q–q plots to evaluate potential deviations from linearity, constant variance, and normality of residuals and random effects. We present our results as mean differences in PHQ-9 scores and 95% confidence intervals (CI).

We conducted four sensitivity analyses. First, we explored whether associations between employment quality and PHQ-9 would differ by changing our definition of low living wage from ∼200% to ∼400% of the federal poverty level in 2021 (*i.e.*, increasing the income threshold from $30,000 to $50,000). Second, we evaluated the minimum strength of an unmeasured confounder to fully explain away the observed associations by calculating the E-value and its lower bound for our primary model (model 2).^32^ The formula for the E-value is: $E=\exp\left(0.91\frac{\beta }{\sigma_{res}}\right)+\sqrt{\exp\left(0.91\frac{\beta }{\sigma_{res}}\right)\left[\exp\left(0.91\frac{\beta }{\sigma_{res}}\right)-1\right]}$ where β is the unstandardized linear regression coefficient and $\sigma_{res}$ is the residual standard deviation of the outcome obtained from the model.^33^^,^^34^ Third, we explored potential reverse causation, wherein a history of mental health diagnoses (*e.g.,* depression, generalized anxiety disorder) could have systematically decreased employment quality in 2021, by restricting to SGM participants without a self-reported mental health diagnosis in 2021. Fourth, we accounted for potential selection bias due to lost to follow-up using stabilized inverse probability weights. Lost to follow-up was defined as not returning to complete the 2022 and/or 2023 annual questionnaires. Differences between those who remained engaged and those lost to follow-up were initially evaluated using descriptive statistics (Supplementary Table S1). Weights were then constructed using logistic regression as the probability of remaining engaged in 2022 and/or 2023 conditional on employment quality divided by the probability of remaining engaged conditional on socio-demographic characteristics, history of mental health diagnosis, employment quality, and PHQ-9 scores in 2021. Distribution of these stabilized weights were used to assess potential model misspecification and is shown in Supplementary Figure S1.

Two post-hoc analyses were performed: (i) pairwise comparisons of PHQ-9 scores across all employment quality groups using Model 2 and (ii) separate models examining the association of annual income and employment status with PHQ-9 to examine their contributions to PHQ-9 scores over the study period. All analysis were conducted in R version 4.3.1. Mixed-effects models were fitted using *lme4,* E-values were calculated using *E-Value*, and weights were generated using *WeightIt*.^32^^,^^35^^,^^36^

### Role of the funding source

A philanthropic gift was the primary funding source that provided salary support of the first author, research staff, and research-related expenses. The PRIDE Study's digital infrastructure was partially funded by the Patient-Centered Outcomes Research Institute (PCORI) [award number PPRN-1501-26848]. Funders did not have a role in the design and conduct of the study; collection, management, analysis, and interpretation of the data; preparation, review, or approval of the manuscript; or decision to submit the manuscript for publication.

## Results

**Fig. 1:**
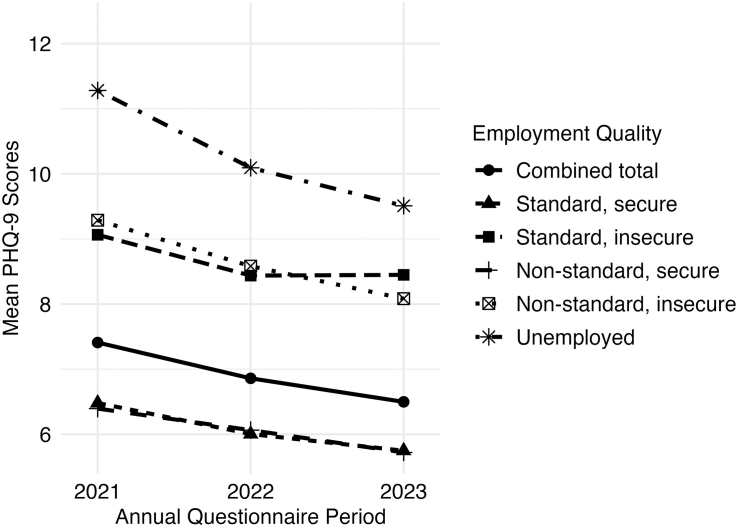
**Trajectories in mean PHQ-9 scores over time by employment quality in The PRIDE Study, 2021**–**2023**. Abbreviation: PHQ-9, Patient Health Questionnaire-9.

The study population consisted of 4897 participants who completed the 2021 questionnaire. Of those, we excluded participants who were not in the labor market (n = 982), students (n = 554), and those missing data on employment (n = 3) and gender identity (n = 4), resulting in a final sample of 3354 SGM participants with 7940 observations. The median age of participants was 34.7 years (interquartile range, 28.8–44.8; Table 1). Most participants self-identified as white, including white and another race/ethnicity (91.5%; n = 3070), followed by Hispanic or Latino (6.2%; n = 208). Other ethnoracial identities were selected by fewer than 5% of the sample. Most participants were undergraduate- or graduate-level educated (83.0%; n = 2782), US-born (95.6%; n = 3206), and living in an urban area (92.7%; n = 3108). The sample was 53.1% (n = 1782) cisgender sexual minority participants and 46.9% (n = 1572) TGD participants. Mean PHQ-9 scores for the overall sample showed a slight decline from 7.4 in 2021 to 6.5 in 2023 (Fig. 1, Supplementary Table S2).

**Table 1:** Participants characteristics by employment quality in The PRIDE Study at baseline, 2021–2023.

	Total	Standard, secure-income employment	Standard, insecure-income employment	Non-standard, secure-income employment	Non-standard, insecure-income employment	Unemployment
	n = 3354 (100)	n = 1913 (57.0)	n = 313 (9.3)	n = 432 (12.88)	n = 500 (14.9)	n = 196 (5.8)
Age (median, IQR)	34.7 (28.8–44.8)	35.7 (30.3–45.2)	28.1 (24.3–32.8)	40.5 (32.3–58.3)	31.4 (25.3–42.7)	29.8 (24.2–38.6)
Gender Identity^a^ (n, %)						
Agender	176 (5.2)	79 (4.1)	23 (7.3)	15 (3.5)	47 (9.4)	12 (6.1)
Cisgender man	619 (18.5)	429 (22.4)	33 (10.5)	78 (18.1)	44 (8.8)	35 (17.9)
Cisgender woman	843 (25.1)	515 (26.9)	73 (23.3)	112 (25.9)	108 (21.6)	35 (17.9)
Genderqueer	474 (14.1)	210 (11.0)	56 (17.9)	56 (13.0)	110 (22.0)	42 (21.4)
Man	661 (19.7)	384 (20.1)	49 (15.7)	84 (19.4)	101 (20.2)	43 (21.9)
Non-binary	849 (25.3)	408 (21.3)	105 (33.5)	87 (20.1)	184 (36.8)	65 (33.2)
Questioning	161 (4.8)	85 (4.4)	21 (6.7)	9 (2.1)	30 (6.0)	16 (8.2)
Transgender man	402 (12.0)	174 (9.1)	51 (16.3)	41 (9.5)	99 (19.8)	37 (18.9)
Transgender woman	188 (5.6)	107 (5.6)	20 (6.4)	18 (4.2)	30 (6.0)	13 (6.6)
Two-spirit	22 (0.7)	6 (0.3)	0 (0.0)	5 (1.2)	9 (1.8)	2 (1.0)
Woman	689 (20.5)	400 (20.9)	70 (22.4)	102 (23.6)	87 (17.4)	30 (15.3)
Another gender identity	224 (6.7)	96 (5.0)	30 (9.6)	25 (5.8)	55 (11.0)	18 (9.2)
Missing	0 (0.0)	0 (0.0)	0 (0.0)	0 (0.0)	0 (0.0)	0 (0.0)
Gender Modality (n, %)						
Cisgender	1782 (53.1)	1140 (59.6)	121 (38.7)	267 (61.8)	177 (35.4)	77 (39.3)
Transgender and gender diverse	1572 (46.9)	773 (40.4)	192 (61.3)	165 (38.2)	323 (64.6)	119 (60.7)
Sexual Orientation^a^ (n, %)						
Asexual	335 (10.0)	150 (7.8)	44 (14.1)	35 (8.1)	75 (15.0)	31 (15.8)
Bisexual	1040 (31.0)	542 (28.3)	129 (41.2)	119 (27.5)	181 (36.2)	69 (35.2)
Gay	1160 (34.6)	729 (38.1)	87 (27.8)	159 (36.8)	122 (24.4)	63 (32.1)
Lesbian	744 (22.2)	452 (23.6)	62 (19.8)	97 (22.5)	97 (19.4)	36 (18.4)
Pansexual	557 (16.6)	274 (14.3)	54 (17.3)	77 (17.8)	103 (20.6)	49 (25.0)
Queer	1560 (46.5)	854 (44.6)	156 (49.8)	188 (43.5)	265 (53.0)	97 (49.5)
Questioning	70 (2.1)	29 (1.5)	12 (3.8)	8 (1.9)	13 (2.6)	8 (4.1)
Same-gender loving	142 (4.2)	63 (3.3)	18 (5.8)	17 (3.9)	32 (6.4)	12 (6.1)
Straight	57 (1.7)	31 (1.6)	4 (1.3)	8 (1.9)	10 (2.0)	4 (2.0)
Two-spirit	15 (0.4)	6 (0.3)	0 (0.0)	3 (0.7)	5 (1.0)	1 (0.5)
Another sexual orientation	119 (3.5)	41 (2.1)	19 (6.1)	18 (4.2)	34 (6.8)	7 (3.6)
Missing	0 (0.0)	0 (0.0)	0 (0.0)	0 (0.0)	0 (0.0)	0 (0.0)
Intersex (n, %)						
No	3297 (98.3)	1889 (98.7)	307 (98.1)	425 (98.4)	488 (97.6)	188 (95.9)
Yes	54 (1.6)	22 (1.2)	6 (1.9)	7 (1.6)	11 (2.2)	8 (4.1)
Missing	3 (0.1)	2 (0.1)	0 (0.0)	0 (0.0)	1 (0.2)	0 (0.0)
Ethnoracial identity^a^ (n, %)						
American Indian or Alaska Native	75 (2.2)	31 (1.6)	11 (3.5)	13 (3.0)	15 (3.0)	5 (2.6)
Asian	152 (4.5)	84 (4.4)	19 (6.1)	17 (3.9)	20 (4.0)	12 (6.1)
Black or African American	114 (3.4)	58 (3.0)	11 (3.5)	11 (2.5)	24 (4.8)	10 (5.1)
Hispanic or Latino	208 (6.2)	112 (5.9)	23 (7.3)	22 (5.1)	32 (6.4)	19 (9.7)
Middle Eastern or North African	47 (1.4)	21 (1.1)	5 (1.6)	5 (1.2)	10 (2.0)	6 (3.1)
Native Hawaiian or Pacific Islander	8 (0.2)	4 (0.2)	2 (0.6)	2 (0.5)	0 (0.0)	0 (0.0)
White	3070 (91.5)	1757 (91.8)	284 (90.7)	397 (91.9)	458 (91.6)	174 (88.8)
Another race/ethnicity	45 (1.3)	17 (0.9)	5 (1.6)	7 (1.6)	11 (2.2)	5 (2.6)
Missing	2 (0.1)	1 (0.1)	0 (0.0)	0 (0.0)	1 (0.2)	0 (0.0)
Education level (n, %)						
High school or less	101 (3.0)	18 (0.9)	20 (6.4)	6 (1.4)	38 (7.6)	19 (9.7)
Some college	470 (14.0)	168 (8.8)	65 (20.8)	48 (11.1)	142 (28.4)	47 (24.0)
4-year degree	1207 (36.0)	655 (34.2)	148 (47.3)	118 (27.3)	204 (40.8)	82 (41.8)
Graduate degree	1575 (47.0)	1072 (56.0)	80 (25.6)	260 (60.2)	115 (23.0)	48 (24.5)
Missing	1 (0.0)	0 (0.0)	0 (0.0)	0 (0.0)	1 (0.2)	0 (0.0)
Immigrant status (n, %)						
Non-U.S. born	124 (3.7)	74 (3.9)	7 (2.2)	21 (4.9)	10 (2.0)	12 (6.1)
U.S. born	3206 (95.6)	1828 (95.6)	304 (97.1)	408 (94.4)	482 (96.4)	184 (93.9)
Missing	24 (0.7)	11 (0.6)	2 (0.6)	3 (0.7)	8 (1.6)	0 (0.0)
Region (n, %)						
Northeast	727 (21.7)	419 (21.9)	63 (20.1)	94 (21.8)	105 (21.0)	46 (23.5)
Midwest	677 (20.2)	357 (18.7)	83 (26.5)	83 (19.2)	119 (23.8)	35 (17.9)
South	845 (25.2)	516 (27.0)	96 (30.7)	96 (22.2)	100 (20.0)	37 (18.9)
West	1083 (32.3)	613 (32.0)	71 (22.7)	157 (36.3)	168 (33.6)	74 (37.8)
US territories or military overseas	3 (0.1)	3 (0.2)	0 (0.0)	0 (0.0)	0 (0.0)	0 (0.0)
Missing	19 (0.6)	5 (0.3)	0 (0.0)	2 (0.5)	8 (1.6)	4 (2.0)
Urbanicity (n, %)						
Rural	224 (6.7)	98 (5.1)	32 (10.2)	37 (8.6)	44 (8.8)	13 (6.6)
Urban	3108 (92.7)	1807 (94.5)	281 (89.8)	393 (91.0)	448 (89.6)	179 (91.3)
US territories or military overseas	3 (0.1)	3 (0.2)	0 (0.0)	0 (0.0)	0 (0.0)	0 (0.0)
Missing	19 (0.6)	5 (0.3)	0 (0.0)	2 (0.5)	8 (1.6)	4 (2.0)

Abbreviations: IQR, interquartile range; U.S., United States.

a Participants can select more than one answer choice. As such, these numbers may sum to more than 100%, and do not reflect mutually exclusive categories. 43.1% selected more than one gender identity; 46.8% selected more than one sexual orientation; 9.4% selected more than one race/ethnicity.

Most participants (57.0%; n = 1913) were in the standard, secure-income group; 9.3% (n = 313) in the standard, insecure-income group; 12.8% (n = 432) in the non-standard, secure-income group; 14.9% (n = 500) in the non-standard, insecure-income group; and 5.9% (n = 196) in the unemployed group (Table 1). A higher proportion of the non-standard, insecure-income and unemployed groups were TGD (64.6% [n = 323] and 60.7% [n = 119], respectively) compared to cisgender (35.4% [n = 177] and 39.3% [n = 77], respectively).

**Fig. 2:**
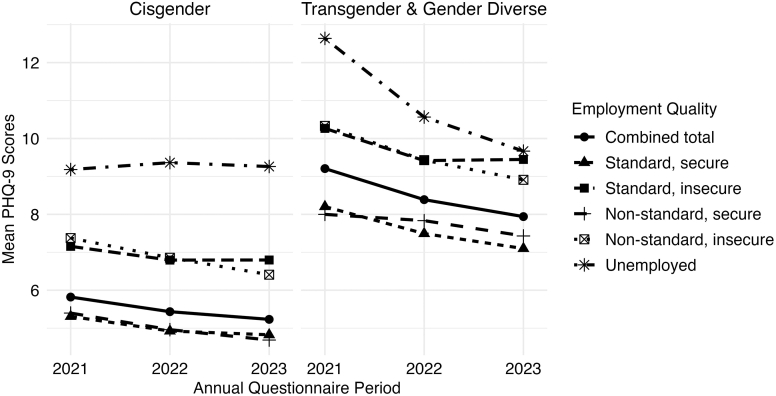
Trajectories in mean PHQ-9 scores over time by employment quality and gender modality in The PRIDE Study, 2021–2023. Abbreviation: PHQ-9, Patient Health Questionnaire-9.

The standard, insecure-income group; non-standard, insecure-income group; and unemployed group reported consistently higher PHQ-9 scores over time compared to those in standard, secure-income group (Fig. 1, Supplementary Table S2). For example, mean PHQ-9 scores of non-standard, insecure-income workers were a minimum of two points higher than standard, secure-income workers in 2021, 2022, and 2023. Mean scores were 3.4–4.5 points higher among unemployed workers in each year compared to standard, secure-income workers. In contrast, PHQ-9 scores were similar between workers in standard, secure-income and non-standard, secure-income groups. When stratified by gender modality, PHQ-9 scores were higher among TGD (compared to cisgender sexual minority) workers in all employment quality groups and were highest among standard, insecure-income; non-standard, insecure-income; and unemployed groups (Fig. 2, Supplementary Table S2). Combining TGD workers in all groups, the mean PHQ-9 score was at least 3 points higher in each year compared to cisgender workers (all groups combined). These patterns were observed across worker groups. To illustrate, mean scores in 2021, 2022, and 2023 for non-standard, insecure-income cisgender workers were 7.4, 6.9, and 6.4, respectively. Whereas for non-standard, insecure-income TGD workers, mean scores were 10.3, 9.4, and 8.9 across years.

Employment quality was associated with PHQ-9 scores in crude and adjusted models (models 1 and 2; Table 2). In an adjusted model (model 2), workers in standard, insecure-income group (mean difference = 0.98; 95% CI, 0.38–1.58; p = 0.0014); non-standard, insecure-income group (mean difference = 1.39; 95% CI, 0.88–1.90; p < 0.0001); and unemployed group (mean difference = 3.12; 95% CI, 2.38–3.85; p < 0.0001) had higher PHQ-9 scores compared to those in the standard, secure-income group. There were also associations for time and gender modality with PHQ-9. For each additional year, PHQ-9 scores declined by 0.35 points (95% CI, −0.44 to −0.26; p < 0.0001). Compared to cisgender participants, TGD participants had higher PHQ-9 scores by 2.12 points (95% CI, 1.77–2.47; p < 0.0001). However, there was no evidence that associations between employment quality and PHQ-9 differed over time (p = 0.28, model 3) or by gender modality (p = 0.76, model 4).

**Table 2:** Associations between employment quality and PHQ-9 scores over time and by gender modality in The PRIDE Study, 2021–2023.

	Model 1		Model 2		Model 3		Model 4	
	Mean difference (95% CI)	p-value	Mean difference (95% CI)	p-value	Mean difference (95% CI)	p-value	Mean difference (95% CI)	p-value
Employment quality								
Standard, insecure-income	2.56 (1.95, 3.18)	<0.0001	0.98 (0.38, 1.58)	0.0014	0.95 (0.10, 1.79)	0.028	0.83 (−0.09, 1.76)	0.08
Non-standard, secure-income	−0.07 (−0.60, 0.47)	0.80	0.44 (−0.08, 0.97)	0.09	0.47 (−0.25, 1.20)	0.20	0.50 (−0.06, 1.25)	0.08
Non-standard, insecure-income	2.62 (2.12, 3.13)	<0.0001	1.39 (0.88, 1.90)	<0.0001	1.75 (1.04, 2.46)	<0.0001	1.48 (0.68, 2.28)	0.0003
Unemployed	4.52 (3.76, 5.28)	<0.0001	3.12 (2.38, 3.85)	<0.0001	3.79 (2.76, 4.83)	<0.0001	3.59 (2.46, 4.72)	<0.0001
Time (years)	−0.36 (−0.45, −0.27)	<0.0001	−0.35 (−0.44, −0.26)	<0.0001	−0.30 (−0.42, −0.18)	<0.0001	−0.35 (−0.44, −0.26)	<0.0001
Employment Quality × Time								
Standard, insecure-income × Time					0.02 (−0.31, 0.35)	0.89		
Non-standard, secure-income × Time					−0.02 (−0.29, 0.25)	0.88		
Non-standard, insecure-income × Time					−0.20 (−0.47, 0.07)	0.15		
Unemployed × Time					−0.37 (−0.78, 0.03)	0.07		
TGD			2.12 (1.77, 2.47)	<0.0001	2.12 (1.77, 2.47)	<0.0001	2.22 (1.77, 2.67)	<0.0001
Employment quality × TGD								
Standard, insecure-income × TGD							0.20 (−0.99, 1.40)	0.74
Non-standard, secure-income × TGD							−0.39 (−1.43, 0.65)	0.46
Non-standard, insecure-income × TGD							−0.18 (−1.19, 0.84)	0.73
Unemployed × TGD							−0.82 (−2.29, 0.66)	0.28
AIC	46372.5		45,417.1		45420.0		45423.2	
Log-Likelihood	−23176.2		−22688.5		−22686.0		−22687.6	
Likelihood ratio Test p-value					0.28		0.76	
Number of participants	3354		3307		3307		3307	
Number of observations	7940		7847		7847		7847	

Abbreviations: AIC, Akaike Information Criterion; CI, confidence interval; PHQ-9, Patient Health Questionnaire-9; TGD, transgender and gender diverse.

In addition to time and gender modality, models 2, 3, and 4 were adjusted for grand mean-centered age, education levels, immigration status, urbanicity, and region.

Sensitivity analyses suggested results were robust to potential sources of bias (Supplementary Table S3). Increasing the income threshold or applying stabilized inverse probability weights resulted in estimates consistent with primary results. Restricting the sample to those without a prior history of mental health diagnosis slightly attenuated associations for the non-standard, insecure-income (mean difference = 0.87; 95% CI, 0.17–1.57; p = 0.015) and unemployed groups (mean difference = 2.65; 95% CI, 1.53–3.78; p < 0.0001). The E-values for standard, insecure-income (2.07); non-standard, insecure-income (2.48); and unemployed (4.81) groups suggested that an unmeasured confounder would need to be associated with employment quality and PHQ-9 score by a risk ratio of at least two-fold beyond the measured covariates to fully explain away the results.

Post-hoc pairwise comparisons indicated that unemployed participants had higher PHQ-9 scores compared to workers in the standard, insecure-income group (mean difference = 2.14; 95% CI, 1.26, 3.01; p < 0.0001); non-standard, secure-income group (mean difference = 2.68; 95% CI, 1.84, 3.53; p < 0.0001); and non-standard, insecure-income group (mean difference = 1.73; 95% CI, 0.92, 2.54; p < 0.0001; Supplemental Table S4). Similar differences in PHQ-9 scores comparing insecure to secure income were observed for standard and non-standard employment. For post-hoc analysis examining annual income and employment status in separate models (Supplemental Table S5), participants with insecure income (≤$30,000) had PHQ-9 scores that were 0.98 points higher (95% CI, 0.58, 1.39, p < 0.0001) compared to those with secure income. Participants in non-standard employment had PHQ-9 scores that were 0.78 points higher (95% CI, 0.40, 1.17, p < 0.0001) compared to those in standard employment while those who were unemployed had higher PHQ-9 scores compared to workers in standard employment (mean difference = 2.89; 95% CI, 2.17, 3.62; p < 0.0001) and non-standard employment (mean difference = 2.11; 95% CI, 1.35, 2.87, p < 0.0001).

## Discussion

Our study highlights the importance of employment and employment quality in SGM mental health inequities.^19^^,^^37^, ^38^, ^39^, ^40^ We provided important descriptions of employment quality among SGM workers and identified that standard, insecure-income employment; non-standard, insecure-income employment; and unemployment were associated with greater depressive symptoms. Unadjusted and adjusted models demonstrated more severe symptoms across progressively poorer employment quality with substantially stronger, clinically meaningful associations with unemployment relative to other employment quality groups.^27^ However, there was no indication that the association between employment quality and depressive symptoms changed over time. TGD workers reported poorer employment quality and higher depressive symptoms than cisgender sexual minority workers.

Our findings suggest that unemployment is associated with a substantially higher burden of depressive symptoms relative to both standard and non-standard employment, regardless of income security, while income insecurity itself appears to exert an additional and consistent negative effect on depressive symptoms across employment quality groups. Further, we illuminated the combined effects of income and employment status by using a multidimensional measure of employment quality showing a stronger combined association than either one measured independently. Building on prior research, we showed that income is a key element of employment quality that remains overlooked in health research.^9^

We considered employment quality as a social determinant of health at the employer relationship level rather than interpersonal workplace level. The employer relationship (*e.g.*, standard *versus* non-standard employment) shapes interpersonal workplace dynamics; both potentially impacting depression in different ways.^12^^,^^39^ At the interpersonal level, foundational minority stress theory focused on heteronormative workplaces where cisgender gay men faced minority stress, a concept commonly employed to explain health disparities among SGM people.^17^ Workplace discrimination is associated with suicidality and depression and is more common among insecure-income workers.^37^ Importantly, employment non-discrimination protections have been demonstrated to improve mental health among SGM workers.^41^ At the employer relationship level in our study, poorer employment quality (including unemployment) was associated with more severe depressive symptoms, with financial insecurity likely being an important contributor to these differences.^39^^,^^40^ Furthermore, workers in non-standard, insecure-income employment may not have adequate avenues to address workplace discrimination due to fear of retaliation, which may contribute to poorer mental health.^40^^,^^41^ Not having avenues to address discrimination, financial insecurity, and a marginalized employment status (*e.g.*, temporary, unemployed) may explain our findings that non-standard, insecure-income and unemployed SGM workers had higher depressive symptoms. The mechanisms of insecure-income employment, non-standard employment, and unemployment (*e.g.*, financial insecurity, employment uncertainty, increased discrimination in low-quality employment) with higher depressive symptoms among SGM workers require more attention, particularly among workers who are marginalized by their stigmatized identity and their low-quality employment status.^6^

Despite systematic reviews on employment quality demonstrating an impact on mental health, SGM workers have been almost completely overlooked.^8^^,^^42^ Our study addresses this gap with a multidimensional understanding of employment quality and analysis of a large, national sample that is diverse in age, geographic location, gender identity, and sexual orientation.^9^^,^^43^ The longitudinal design is a notable strength; findings align with a systematic review in which studies with one-year follow-up were able to see associations between low-quality employment and depression.^7^ We did not observe a significant interaction with depressive symptoms over time in our study, which might suggest that employment quality was associated with persistently elevated symptoms and not with increased depressive symptom severity. This may speak to employment quality as a more chronic exposure such that elevated depressive symptoms reflect the accumulation of poor labor market conditions rather than immediate changes to a persons’ employment quality.^39^ Given the divergence in our findings and those of general population studies, more longitudinal research among SGM populations is needed to better understand this finding.

Prior studies are subjected to measurement error of employment quality as a construct because of single-item exposures (*e.g.*, temporary employment).^9^ We aimed to address this (and provide guidance for future research) by employing indicators of employment quality that are often associated with other employment quality indicators. For example, those working 40+ hours per week on a permanent basis and earning over $30,000 would be more likely to have characteristics of standard employment (*e.g.*, health insurance). Those with low incomes and in part-time or temporary employment are more likely to not have workplace benefits such as health insurance, even though these specific characteristics (*i.e.*, health insurance) were not explicitly explored in our analysis. However, without a robust variable set inclusive of subjective (*e.g.*, job satisfaction) and objective (*e.g.*, employment contract) measures, our measure may be imperfect.

This study has additional limitations. Given the lack of sexual orientation and gender identity data collection in national surveys that consider employment and health variables, our reliance on a convenience sample may impact generalizability. Results may also be subject to sampling and recall bias. Potential misclassification of employment quality may have occurred due to the use of self-reported income based on the 2020 tax year to categorize employment quality in 2021, thus not reflecting participants’ 2021 circumstances if their income changed following the 2020 tax year. The magnitude of association was small for standard, insecure-income and non-standard, insecure-income groups. However, the direction of these relationships aligned with extant literature.^8^^,^^43^ Our sample was highly educated. People with higher levels of education may have been more likely to participate in our study and, among these individuals, those in low-quality employment or unemployed may not experience the same level of distress as those who are in low-quality employment with limited education. Limited employment variables were available within our study, and future studies should explore specific characteristics of employment quality related to the employer relationship, including health insurance, unionization, job satisfaction, and autonomy. Ethnoracial minorities face increased workplace discrimination and insecure-income employment.^37^ However, due to the limited ethnoracial diversity in our study, we were unable to conduct these intersectional analyses. The aim of this study was to examine the associations of a multidimensional measure of employment quality with depressive symptoms. However, future research might consider income and hours worked separately in their association, as well as how much income is needed to support mental health. Further stratification among non-standard workers may also find important differences between workers with varying levels of precarity. Our sensitivity analyses indicate that impact on depressive symptoms were similar for $30,000 and $50,000 cut offs; however, people earning above $50,000 may face economic insecurity. Therefore, people grouped as “secure-income” in our sample may still experience varying degrees of economic insecurity.

For SGM workers, poor employment quality can be related to adverse mental health symptoms. Interventions should address mental health, employment quality, and income among SGM workers. Reducing the number of workers exposed to insecure-income employment and unemployment may reduce the burden of depression among SGM workers. These findings should motivate SGM health researchers to consider employment as a central variable in SGM mental and physical health inequities research.

## Contributors

Each author has made significant and meaningful contributions to this manuscript. DJK led conceptualization, project administration, and writing of this manuscript. NKT supported conceptualization and led the analysis. FVS consulted on conceptualization of the exposure and execution of the analysis. SS and KBB provided consultation on the interpretation. AF supervised DJK and provided insights into conceptualization of the outcomes. MEL led data collection through participant recruitment. JOM, MRL, and AF secured funding for the study, led broader project administration, and supervised the study. MRL supervised DJK throughout the study, informing all aspects of the manuscript preparation. MRL, DJK, and NKT all directly accessed and verified the underlying data reported in the manuscript. All authors provided review and editing of the manuscript. DJK made the decision to submit the manuscript for publication.

## Data sharing statement

Data underlying the study cannot be made publicly available due to ethical concerns about participant confidentiality. Researchers interested in The PRIDE Study data can submit a brief application to be reviewed by a Research Advisory Committee (composed of scientists) and Participant Advisory Committee (composed of participants) to affirm appropriate data use.

## Declaration of interests

Dr. Juno Obedin-Maliver has consulted for Ibis Reproductive Health (2020-present), Hims and Hers Health, Incorporated (2019-present), Folx Health, Incorporated (2019-present), and Upstream, Incorporated (2024). Dr. Lunn has consulted for Otsuka Pharmaceutical Development and Commercialization, Incorporated (2023) and the American Dental Association (2024). None of these engagements influenced or are pertinent to the work described in this manuscript.
