# Supplementary material for: Association of employment quality with depression among sexual and gender minority adults: a retrospective cohort study
Source: Lancet Reg Health Am. 2026 Mar 26;58:101462. doi: 10.1016/j.lana.2026.101462 (PMC13050029; doi:10.1016/j.lana.2026.101462)
Supplement: Supplementary Material [file mmc1.docx]

**Supplementary Material**

Kinitz DJ, Tran NK, Shahidi FV, Soltani S, Bryant-Lees KB, Flentje A, Lubensky ME, Obedin-Maliver J, Lunn MR. Longitudinal association of employment quality with depression in a sexual and gender minority adult cohort.

**Table of Contents**

**Page 2: Supplemental Table 1.** Characteristics of participants by engagement status in The PRIDE Study, 2021-2023.

**Page 3: Supplemental Figure 1.** Distribution of censoring (lost to follow-up in 2022 and 2023) weights in The PRIDE Study.

**Page 4: Supplemental Table 2.** PHQ-9 scores over the study period by employment quality and gender modality in The PRIDE Study, 2021-2023.

**Page 5: Supplemental Table 3.** Sensitivity analyses of the adjusted association between employment quality and depressive symptoms in The PRIDE Study, 2021-2023.

**Page 6: Supplemental Table 4.** Pairwise differences in PHQ-9 scores between employment quality group in The PRIDE Study, 2021-2023.

**Page 7: Supplemental Table 5.** Mean differences in PHQ-9 scores by annual income and employment status in The PRIDE Study, 2021-2023.

This supplemental material has been provided by the authors to give readers additional information about their work.

**Supplemental Table 1. Characteristics of participants by engagement status in The PRIDE Study, 2021-2023.**

|  | Remained engaged | Lost to follow-up |
| --- | --- | --- |
|  | (n = 2618) | (n = 736) |
| Age (median, IQR) | 35·0 (29·1-45·4) | 33·4 (27·8-43·3) |
| Gender Modality (n, %) |  |  |
| Cisgender | 1372 (52·4) | 410 (55·7) |
| Transgender and gender diverse | 1246 (47·6) | 326 (44·3) |
| Education level (n, %) |  |  |
| High school or less | 68 (2·6) | 33 (4·5) |
| Some college | 338 (12·9) | 132 (17·9) |
| 4-year degree | 932 (35·6) | 275 (37·4) |
| Graduate degree | 1279 (48·9) | 296 (40·2) |
| Missing | 1 (0·0) | 0 (0·0) |
| Immigrant status (n, %) |  |  |
| Non-U.S. born | 95 (3·6) | 29 (3·9) |
| U.S. born | 2512 (96·0) | 694 (94·3) |
| Missing | 11 (0·4) | 13 (1·8) |
| Region (n, %) |  |  |
| Northeast | 558 (21·3) | 169 (23·0) |
| Midwest | 528 (20·2) | 149 (20·2) |
| South | 655 (25·0) | 190 (25·8) |
| West | 860 (32·8) | 223 (30·3) |
| Other (U.S. possessions or military overseas) | 2 (0·1) | 1 (0·1) |
| Missing | 15 (0·6) | 4 (0·5) |
| Urbanicity (n, %) |  |  |
| Rural | 182 (7·0) | 42 (5·7) |
| Urban | 2419 (92·4) | 689 (93·6) |
| Other (U.S. possessions or military overseas) | 2 (0·1) | 1 (0·1) |
| Missing | 15 (0·6) | 4 (0·5) |
| Employment quality (n, %) |  |  |
| Standard, secure-income | 1492 (57·0) | 421 (57·2) |
| Standard, insecure-income | 235 (9·0) | 78 (10·6) |
| Non-standard, secure-income | 355 (13·6) | 77 (10·5) |
| Non-standard, insecure-income | 385 (14·7) | 115 (15·6) |
| Unemployed | 151 (5·8) | 45 (6·1) |
| History of mental health diagnosis in 2021 (n, %) | 1768 (67·5) | 500 (67·9) |
| PHQ-9 score in 2021 (mean, SD) | 7·3 (5·8) | 8·0 (6·2) |

Abbreviations: IQR, interquartile range; GAD-7, Generalized Anxiety Disorder-7; PHQ-9, Patient Health Questionnaire-9; US, United States.

**Supplemental Figure 1. Distribution of censoring (lost to follow-up in 2022 and 2023) weights in The PRIDE Study.**


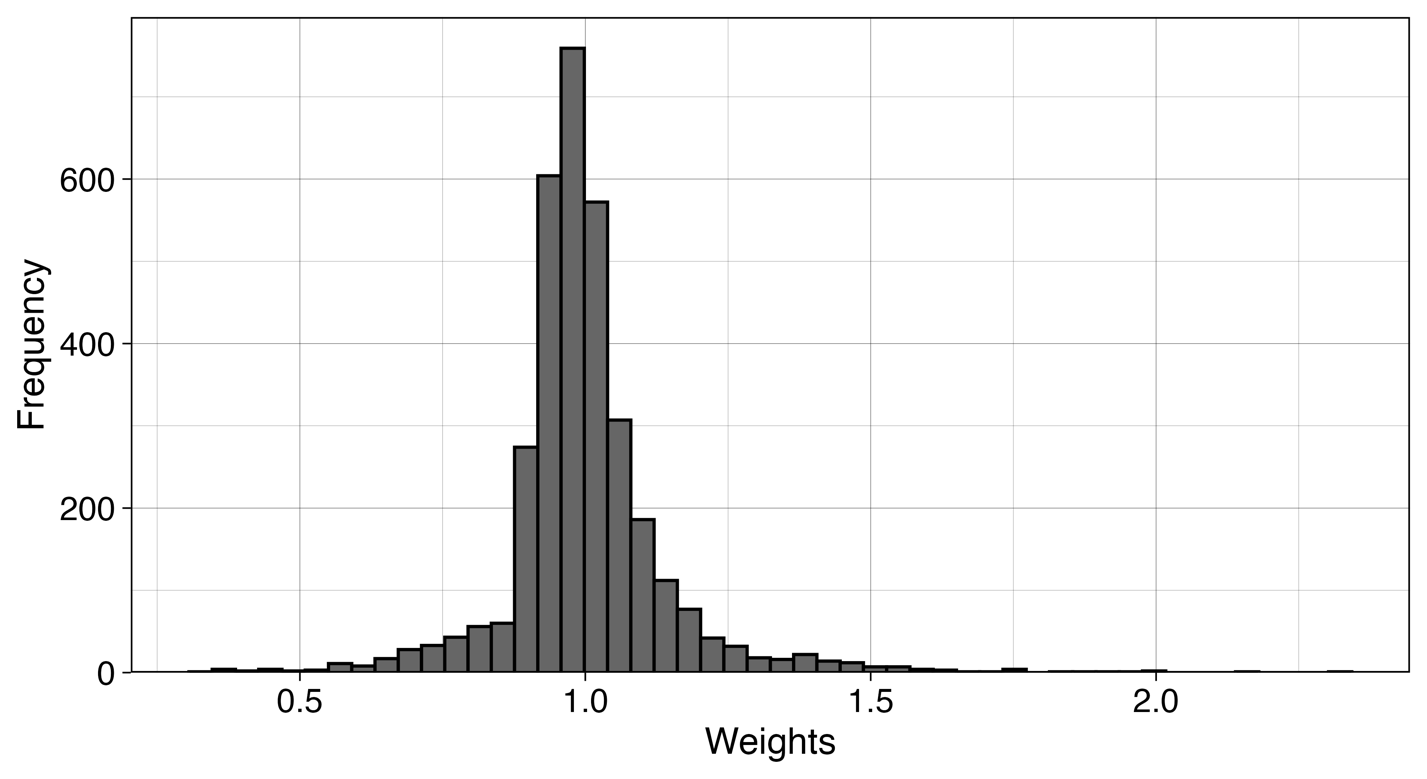


**Supplemental Table 2. PHQ-9 scores over the study period by employment quality and gender modality in The PRIDE Study, 2021-2023.**

|  |  | 2021 | 2022 | 2023 |
| --- | --- | --- | --- | --- |
|  |  | Mean, Median, SD | Mean, Median, SD | Mean, Median, SD |
| **Overall Sample** | | n = 3354 | n = 2411 | n = 2179 |
|  | Combined Total^a^ | 7·4, 6, 5·9 | 6·9, 6, 5·6 | 6·5, 5, 5·6 |
|  | Standard, secure-income | 6·8, 6, 5·6 | 6·3, 5, 5·4 | 6·1, 5, 5·3 |
|  | Standard, insecure-income | 9·1, 8, 5·9 | 8·4, 8, 5·7 | 8·4, 7, 5·9 |
|  | Non-standard, secure-income | 6·4, 5, 5·6 | 6·1, 5, 5·3 | 5·7, 4, 5·2 |
|  | Non-standard, insecure-income | 9·3, 9, 6·1 | 8·6, 8, 5·9 | 8·1, 7, 6·0 |
|  | Unemployed | 11·3, 10, 7·1 | 10·1, 9, 6·4 | 9·5, 8, 6·7 |
| **Cisgender** | | n = 1782 | n = 1248 | n = 1161 |
|  | Combined Total^a^ | 5·8, 5, 5·2 | 5·4, 4, 5·1 | 5·2, 4, 4·8 |
|  | Standard, secure-income | 5·5, 4, 4·9 | 5·1, 4, 4·8 | 5·0, 4, 4·6 |
|  | Standard, insecure-income | 7·2, 6, 5·4 | 6·8, 5, 5·6 | 6·8, 6, 5·4 |
|  | Non-standard, secure-income | 5·4, 4, 5·1 | 5·0, 4, 4·7 | 4·7, 3, 4·7 |
|  | Non-standard, insecure-income | 7·4, 6, 6·0 | 6·9, 6, 5·6 | 6·4, 5·5, 5·0 |
|  | Unemployed | 9·2, 9, 6·7 | 9·4, 8, 6·4 | 9·3, 7, 6·6 |
| **Transgender and Gender Diverse** | | n = 1572 | n = 1163 | n = 1018 |
|  | Combined Total^a^ | 9·2, 8, 6·1 | 8·4, 8, 5·8 | 7·9, 7, 6·0 |
|  | Standard, secure-income | 8·6, 8, 5·8 | 7·9, 7, 5·6 | 7·5, 7, 5·8 |
|  | Standard, insecure-income | 10·3, 10, 5·9 | 9·4, 8, 5·6 | 9·4, 9, 6 |
|  | Non-standard, secure-income | 8·0, 6, 6·0 | 7·8, 7, 5·8 | 7·4, 7, 5·5 |
|  | Non-standard, insecure-income | 10·3, 10, 5·8 | 9·4, 9, 5·9 | 8·9, 8, 6·2 |
|  | Unemployed | 12·6, 12, 7·0 | 10·6, 10, 6·3 | 9·7, 8·5, 6·8 |

Abbreviation: PHQ-9, Patient Health Questionnaire-9.

**Supplemental Table 3. Sensitivity analyses of the adjusted association between employment quality and depressive symptoms in The PRIDE Study, 2021-2023.**

| **Sensitivity analysis** | **Employment quality** | **Mean Difference (95% Confidence Interval)** |
| --- | --- | --- |
| 1. Increase income threshold from 200% to 400% above federal poverty level (n = 3307) | Standard, secure-income | 1.00 (ref) |
|  | Standard, insecure-income | 1·16 (0·71, 1·62), p-value < 0·0001 |
|  | Non-standard, secure-income | 0·47 (-0·19, 1·13), p-value = 0·16 |
|  | Non-standard, insecure-income | 1·49 (1·02, 1·96), p-value < 0·0001 |
|  | Unemployed | 3·43 (2·68, 4·18), p-value < 0·0001 |
|  |  | **Estimate (lower bound)** |
| 1. Calculated E-values for Model 2 to determine minimum strength of an unmeasured confounder   Estimate (lower bound) | Standard, secure-income | Not Applicable |
|  | Standard, insecure-income | 2·07 (1·51) |
|  | Non-standard, secure-income | 1·56 (1·00) |
|  | Non-standard, insecure-income | 2·48 (1·97) |
|  | Unemployed | 4·81 (3·68) |
|  |  | **Mean Difference (95% Confidence Interval)** |
| 1. Restricted to SGM participants to those without a history of mental health diagnoses in 2021 (n = 1071) | Standard, secure-income | 1.00 (ref) |
|  | Standard, insecure-income | 0·99 (0·16, 1·82), p-value = 0·019 |
|  | Non-standard, secure-income | 0·08 (-0·53, 0·68), p-value = 0·81 |
|  | Non-standard, insecure-income | 0·87 (0·17, 1·57), p-value = 0·015 |
|  | Unemployed | 2·65 (1·53, 3·78), p-value < 0·0001 |
|  |  | **Mean Differnce (95% Confidence Interval)** |
| 1. Applied inverse probability of censoring weights to account for differential lost to follow-up (n = 3307) | Standard, secure-income | 1·00 (ref) |
|  | Standard, insecure-income | 0·99 (0·39, 1·59), p-value = 0·0013 |
|  | Non-standard, secure-income | 0·45 (-0·06, 0·96), p-value = 0·09 |
|  | Non-standard, insecure-income | 1·40 (0·89, 1·91), p-value < 0·0001 |
|  | Unemployed | 3·12 (2·39, 3·85), p-value < 0·0001 |

Abbreviation: PHQ-9, Patient Health Questionnaire-9.

**Supplemental Table 4. Pairwise differences in PHQ-9 scores between employment quality group in The PRIDE Study, 2021-2023.**

| **Employment quality comparisons** | **Mean Difference (95% Confidence Interval)** | **p-value** |
| --- | --- | --- |
| Standard, insecure-income - Standard, secure-income | 0·98 (0·38, 1·58) | 0·0014 |
| Non-standard, secure-income - Standard, secure-income | 0·44 (-0·08, 0·97) | 0·09 |
| Non-standard, insecure-income - Standard, secure-income | 1·39 (0·88, 1·90) | <0·0001 |
| Unemployed - Standard, secure-income | 3·12 (2·38, 3·85) | <0·0001 |
| Non-standard, secure-income - Standard, insecure-income | -0·55 (-1·29, 0·20) | 0·15 |
| Non-standard, insecure-income - Standard, insecure-income | 0·41 (-0·29, 1·10) | 0·26 |
| Unemployed - Standard, insecure-income | 2·14 (1·26, 3·01) | <0·0001 |
| Non-standard, insecure-income - Non-standard, secure-income | 0·95 (0·30, 1·61) | 0·0045 |
| Unemployed - Non-standard, secure-income | 2·68 (1·84, 3·53) | <0·0001 |
| Unemployed - Non-standard, insecure-income | 1·73 (0·92, 2·54) | <0·0001 |

Abbreviation: PHQ-9, Patient Health Questionnaire-9.

Model adjusted for time, gender modality, mean-centered age, education levels, immigration status, urbanicity, and region.

**Supplemental Table 5. Mean differences in PHQ-9 scores by annual income and employment status in The PRIDE Study, 2021-2023.**

|  |  | **Mean Difference (95% Confidence Interval)** | **p-value** |
| --- | --- | --- | --- |
| **Annual Income** | |  |  |
|  | Insecure - Secure | 0·98 (0·58, 1·39) | <0·0001 |
| **Employment Status** | |  |  |
|  | Non-standard - Standard | 0·78 (0·40, 1·17) | <0·0001 |
|  | Unemployed - Standard | 2·89 (2·17, 3·62) | <0·0001 |
|  | Unemployed - Non-standard | 2·11 (1·35, 2·87) | <0·0001 |

Abbreviation: PHQ-9, Patient Health Questionnaire-9.

Model adjusted for time, gender modality, mean-centered age, education levels, immigration status, urbanicity, and region.
